# Synthesis and crystal structure of a heterobimetallic cadmium–sodium complex of 1,3,5-triazine-2,4,6-trione, [CdNa_2_(C_3_H_2_N_3_O_3_)_4_(H_2_O)_8_]

**DOI:** 10.1107/S2056989021008148

**Published:** 2021-08-17

**Authors:** R. Divya, B. R. Bijini, V. S. Dhanya, K. Rajendra Babu, M. Sithambaresan

**Affiliations:** a PG Department and Research Centre in Physics, M.G. College, University of Kerala, Thiruvananthapuram 695004, India; b Department of Chemistry, Faculty of Science, Eastern University, Sri Lanka, Chenkalady, Sri Lanka

**Keywords:** crystal structure, heterobimetallic cadmium–sodium complex, gel growth, 1,3,5 triazine-2,4,6-trione, two-dimensional coord­ination polymer

## Abstract

Heterobimetallic crystals of a cadmium–sodium complex of 1,3,5-triazine-2,4,6-trione, namely, μ-aqua-1:2κ^2^ *O*:*O*-hepta­aqua-1κ^3^ *O*,2κ^2^ *O*,3κ^2^ *O*-bis­(μ-4,6-dioxo-1,4,5,6-tetra­hydro-1,3,5-triazin-2-olato)-1:2κ^2^ *O* ^2^:*N* ^1^;2:3κ^2^ *N* ^1^:*O* ^2^-bis­(4,6-dioxo-1,4,5,6-tetra­hydro-1,3,5-triazin-2-olato)-1κ*O* ^2^,3κ*O* ^2^-2-cadmium-1,3-disodium, [CdNa_2_(C_3_H_2_N_3_O_3_)_4_(H_2_O)_8_], were grown by the single gel diffusion technique. The asymmetric unit of the title compound comprises four 1,3,5-triazine-2,4,6-trione ligands, two sodium atoms and one cadmium atom. Of the four ligands, two are monodentately coordinated to two Na atoms. The third ligand is coordinated bidentately to one Na and the Cd atom and the fourth is also coordinated bidentately to the Cd atom and the other Na atom. All the metal atoms are six-coordinate with a distorted octa­hedral geometry. The water mol­ecules bridge the Na atoms, constructing coordination polymer chains along the *a* axis and hence are linked by two Cd and one Na coordinations through the cyanuric acid ligands present in the coordination polymer chains, generating a two-dimensional coordination polymer in the (110) plane. The polymer formation is further assisted by means of many inter­molecular and intra­molecular N—H⋯O, O—H⋯O and O–H⋯N hydrogen bonds between the water mol­ecules and the ligands.

## Abstract 2

In the heterobimetallic cadmium–sodium complex, hepta­aqua-1κ^3^ *O*,2κ^2^ *O*,3κ^2^ *O*-bis­(μ-4,6-dioxo-1,4,5,6-tetra­hydro-1,3,5-triazin-2-olato)-1:2κ^2^ *O* ^2^:*N* ^1^;2:3κ^2^ *N* ^1^:*O* ^2^-bis­(4,6-dioxo-1,4,5,6-tetra­hydro-1,3,5-triazin-2-olato)-1κ*O* ^2^,3κ*O* ^2^-2-cadmium-1,3-disodium, the ligand coordination around the Cd and Na atoms leads to the formation of a two-dimensional coordination polymer in the (110) plane, which is supported by means of a variety of N—H⋯O, O—H⋯O and O—H⋯N inter­molecular and intra­molecular inter­actions owing to different substitution patterns.

## Chemical context   

Chelation is considered as the preferred method for the reduction of toxic effects of heavy metals, in which the metals are removed in the form of stable complex chelates. Cadmium, one of the most toxic heavy metals, can accumulate in the human body, leading to renal dysfunction, lung cancer, *etc*. In addition, chelation reactions are utilized in the determination of cadmium toxicity (Flora & Pachauri, 2010 ▸) with 1,3,5-triazine-2,4,6-trione, also known as cyanuric acid, being the preferred ligand used for the chelation as it has multiple hydrogen-bond donor centres (Mistri *et al.*, 2014 ▸). 1,3,5-Triazine-2,4,6-trione exists in either the keto or enol form but the most stable isomer is the keto form (Reva, 2015 ▸). In this work, we report the crystal structure of a heterobimetallic cadmium and sodium complex of 1,3,5-triazine-2,4,6-trione.

## Structural commentary   

**Figure 1:**
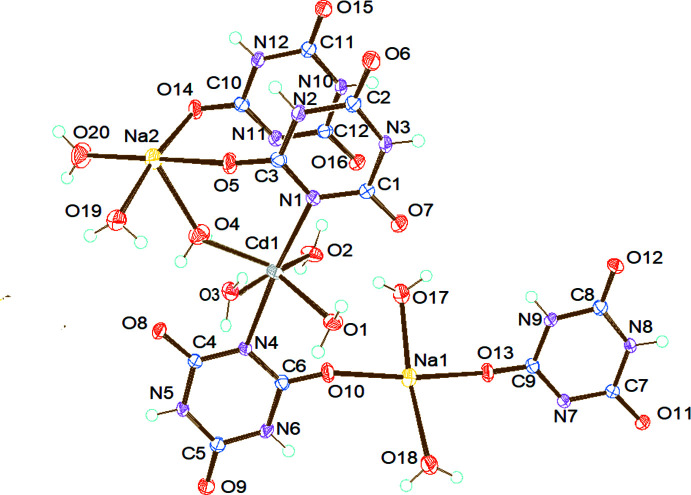
The asymmetric unit of the title compound with displacement ellipsoids drawn at the 50% probability level.

The title complex crystallizes in the triclinic space group *P* 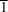. Fig. 1 ▸ shows the asymmetric unit of the crystal, which consists of four cyanuric acid ligands, two sodium atoms (Na1 and Na2) and one cadmium atom. Of the four ligands, two are monodentately coordinated to Na1 and Na2 atoms each. The third ligand is coordinated bidentately to Na1 and Cd1 atoms and the fourth one also coordinated bidentately to Na2 and Cd1 atoms. The sodium atom Na2 is coordinated to oxygen atoms of two cyanuric acid ligands [O5—Na2—O14 = 94.52 (6)°]. The Na1 atom is also coordinated to oxygen atoms of two cyanuric acid ligands [O10—Na1—O13 = 173.39 (7)°]. The Cd1 atom is coordinated to nitro­gen atoms of two cyanuric acid ligands [N1—Cd1—N4 = 174.58 (6)]°. In addition to the ligand coordination, atoms Na1, Na2 and Cd1 are also coordinated by two, three and four water mol­ecules, respectively.
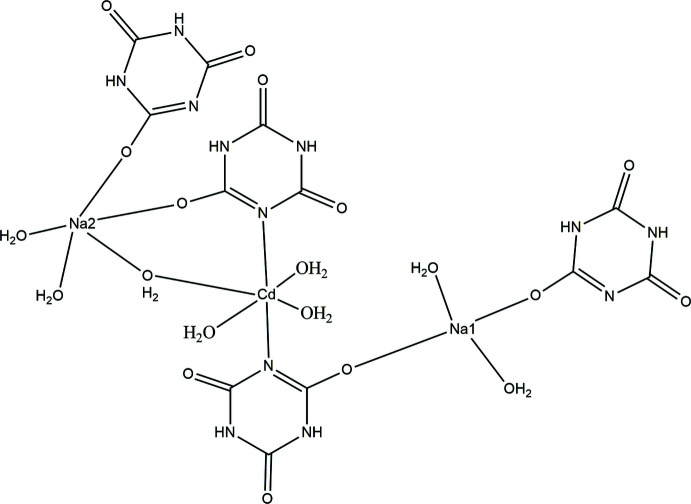


The title compound forms a two dimensional coordination polymer. In the coordination environment of the polymer, the Na1 atom is six-coordinate with the coordination angle varying from 90° [78.82 (6)–90.60 (6)°], forming a distorted octa­hedral geometry. Atom Na2 also exhibits a distorted octa­hedral geometry, with the coordin­ation angles ranging from 77.17 (6) to 91.77 (6)°. The cadmium atom also shows a distorted octa­hedral geometry with coordination angles in the range 87.94 (5) to 95.46 (6)°. A similar geometry is observed for the Cd atom in the heterobimetallic compound tetra­aqua­bis­(malonato)cadmium(II)copper(II) (Dhanya *et al.*, 2014 ▸). Na1—O bond distances [2.3471 (16) to 2.4195 (18) Å] show a decrease compared to the value reported for a free Na—O bond (2.421 Å; Brown & Shannon, 1973 ▸). The Na2—O bonds, which vary from 2.3067 (17) to 2.4997 (18) Å, are longer than the reported value for a free Na—O bond. This observed range of Na—O bonds also shows close agreement with values reported for sodium 2-amino­terephthalate where the sodium atom adopts a similar six-coordinate geometry (2.326–2.505 Å; Sienkiewicz-Gromiuk *et al.*, 2012 ▸). The Cd1—N4 bond distance [2.3210 (15) Å] is comparable to the reported value in a similar coordinated geometry (2.324 Å; Hashemian & Mangeli, 2017 ▸). The water mol­ecules are tetra­hedrally coordinated to the cadmium atom, forming bond angles ranging from 81.59 (5) to 111.45 (5)°. Three water mol­ecules are coordinated to Na2, with bond angles ranging from 85.43 (6) to 102.25 (7)°. Two water mol­ecules are coordinated to Na1, forming a bond angle of 170.94 (7)°. The two water mol­ecules coordinated to Na1 bridge adjacent Na1 atoms on both sides, forming a coord­in­ation polymer chain along the *a* axis. These chains are inter­connected by means of two Cd1 and Na2 coordinations through the cyanuric acid ligands present in the Na1 coordination polymer chain on both sides to build a 2D coordination polymer in the (110) plane.

## Supra­molecular features   

**Figure 2:**
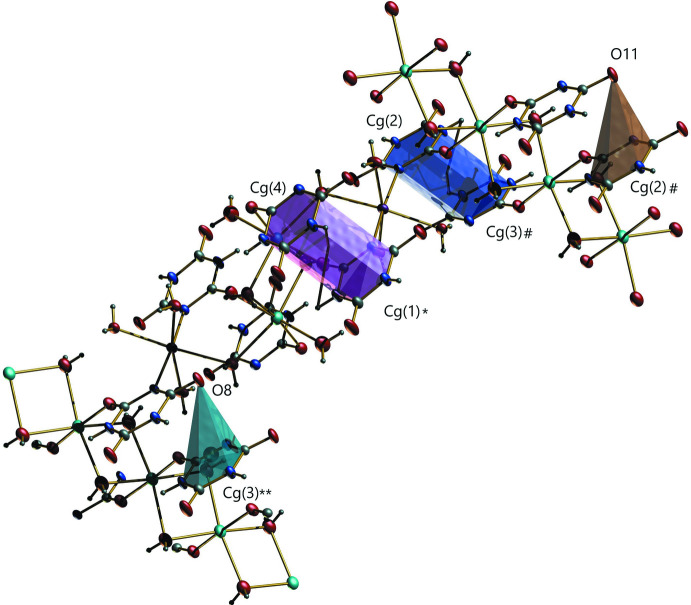
π⋯π and C—O⋯π inter­actions present in the complex. *Cg*1, *Cg*2, *Cg*3 and *Cg*4 are the centroids of the N1–N3/C1–C3, N4–N6/C4–C6, N7–N9/C7–C9 and N10–N12/C10–C12 rings, respectively. Symmetry codes: (*) *x* − 1, *y*, *z*; (**) 1 − *x*, −*y*, −*z*; (#) 2 − *x*, −*y*, −*z*.

**Figure 3:**
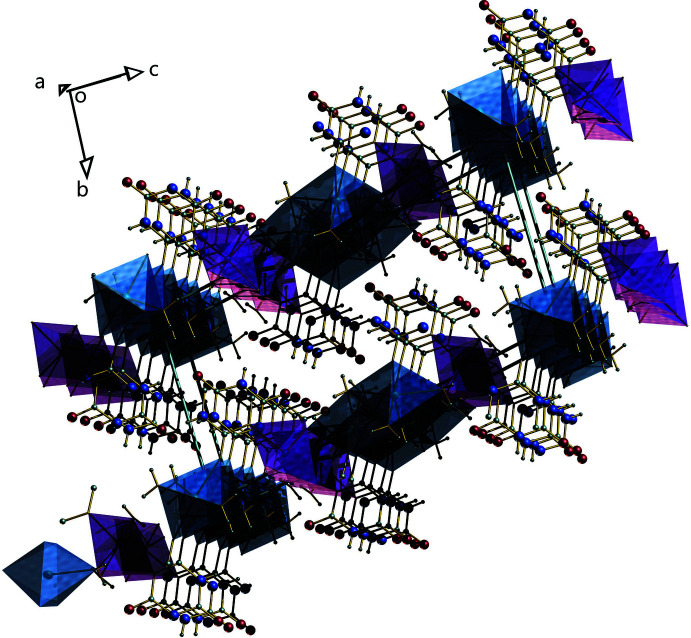
The packing, viewed down the *a* axis, showing the coordination polyhedra.

There are 25 intra­molecular and inter­molecular hydrogen-bonding inter­actions involving the ligands and the coordinated water mol­ecules with *D*⋯*A* distances ranging from 2.716 (2) to 3.236 (2) Å (Table 1 ▸). Two types of π–π inter­actions (Table 2 ▸) occur between the cyanurate rings of different units, having centroid–centroid distances of 3.5174 (12) and 3.4893 (11) Å, and two types of C—O⋯π inter­actions (Table 3 ▸) with different cyanurate rings with *X*⋯*Cg* distances of 3.6086 (2) and 3.4783 (2) Å are also present in the complex (Fig. 2 ▸). A packing diagram is presented in Fig. 3 ▸.

## Synthesis and crystallization   

Needle-shaped transparent single crystals were obtained by the single gel diffusion method (Chandran *et al.*, 2017 ▸). 1,3,5-Triazine-2,4,6-trione, acetic acid, sodium metasilicate and cadmium chloride hydrate were used for the growth in a single glass test tube of length 20 cm and diameter 2.5 cm. The preparation of silica gel of specific gravity 1.03–1.05 g cm^−3^ involved dissolution of sodium metasilicate (SMS) in double-distilled water to which 1,3,5-triazine-2,4,6-trione (0.01–0.02 *M* concentration) was added. The resulting SMS solution was acidified with drops of glacial acetic acid to adjust the pH to within the range 4–7. The test tubes were filled with 30 ml of the above solution for gel setting. Over the set gel, cadmium chloride solution (0.25–1 *M*) was added carefully along the sides of the test tube to prevent the gel breakage. Finally, the test tube was sealed with a transparent plastic sheet to prevent contamination and kept undisturbed for crystal growth. Crystals formed within the gel after two weeks and growth was completed in a month. A series of trials was undertaken to obtain the optimum conditions to grow well-defined single crystals. 1,3,5-Triazine-2,4,6-trione (0.02 *M*) was used as inner reactant and cadmium chloride (0.25 *M*) as the top solution. Well-defined good-quality single crystals suitable for single-crystal XRD studies were grown in a gel medium of pH 6 and density 1.03 g cm^−3^.

## Refinement   

Crystal data, data collection and structure refinement details are summarized in Table 4 ▸. Some reflections truncated by beamstop were omitted. The nitro­gen-bound H atoms were placed in calculated positions (N—H = 0.86 Å) and were included in the refinement in the riding-model approximation, with *U*_iso_(H) set to 1.2*U*_eq_(N). H atoms attached to water mol­ecules were located from difference-Fourier maps and were refined with isotropic displacement parameters [*U*_iso_(H) = 1.5*U*_eq_(O)].

## Supplementary Material

Crystal structure: contains datablock(s) I. DOI: 10.1107/S2056989021008148/jy2009sup1.cif

Structure factors: contains datablock(s) I. DOI: 10.1107/S2056989021008148/jy2009Isup2.hkl

CCDC reference: 1576691

Additional supporting information:  crystallographic information; 3D view; checkCIF report

## Figures and Tables

**Table 1:** Hydrogen-bond geometry (Å, °)

*D*—H⋯*A*	*D*—H	H⋯*A*	*D*⋯*A*	*D*—H⋯*A*
O1—H1*A*⋯N11^i^	0.98	1.82	2.788 (2)	168
O1—H1*B*⋯N7^ii^	0.98	1.89	2.860 (2)	169
N2—H2⋯O15^iii^	0.86	1.96	2.817 (2)	173
O2—H2*A*⋯O13^iv^	0.98	1.77	2.716 (2)	161
O2—H2*B*⋯N11	0.98	2.53	3.236 (2)	128
O2—H2*B*⋯O16	0.98	1.79	2.753 (2)	167
N3—H3⋯O11^v^	0.86	1.92	2.773 (2)	173
O3—H3*A*⋯O14^i^	0.98	1.91	2.871 (2)	166
O3—H3*B*⋯O11^ii^	0.98	1.87	2.842 (2)	169
N5—H5⋯O16^vi^	0.86	2.10	2.938 (2)	164
N6—H6⋯O12^vi^	0.86	2.13	2.979 (2)	168
N10—H10⋯O8^vii^	0.86	1.94	2.792 (2)	172
N8—H8⋯O7^v^	0.86	2.02	2.862 (2)	167
N12—H12⋯O6^iii^	0.86	2.03	2.882 (2)	173
N9—H9⋯O9^vii^	0.86	2.04	2.885 (2)	169
O20—H20*B*⋯O6^viii^	0.98	2.30	2.903 (2)	119
O20—H20*A*⋯O14^ix^	0.98	2.14	3.008 (2)	147
O4—H4*A*⋯O8	0.98	1.88	2.725 (2)	143
O17—H17*B*⋯O2	0.99	1.87	2.768 (2)	149
O17—H17*A*⋯O9^vii^	0.98	1.81	2.790 (2)	171
O18—H18*B*⋯O12^vi^	0.98	1.91	2.852 (2)	160

Symmetry codes: (i) x+1, y, z; (ii) -x+2, -y, -z; (iii) -x+1, -y+1, -z+1; (iv) -x+1, -y, -z; (v) -x+2, -y+1, -z; (vi) x, y-1, z; (vii) x, y+1, z; (viii) x-1, y-1, z; (ix) -x, -y, -z+1.

**Table 2:** Analysis of π–π inter­actions (Å, °) α is the dihedral angle between planes *I* and *J. Cg*1, *Cg*2, *Cg*3 and *Cg*4 are the centroids of the N1–N3/C1–C3, N4–N6/C4–C6, N7–N9/C7–C9 and N10–N12/C10–C12 rings, respectively.

*Cg*(*I*)⋯*Cg*(*J*)	*Cg*⋯*Cg*	α
*Cg*1⋯*Cg*4^i^	3.5174 (12)	2.42 (10)
*Cg*2⋯*Cg*3^ii^	3.4893 (11)	2.59 (9)

Symmetry codes: (i) 1 + *x*, *y*, *z*; (ii) 2 − *x*, −*y*, −*z*.

**Table 3:** Analysis of *Y*—*X*⋯*Cg* (π-ring) inter­actions (Å, °)

*Y*—*X*(*I*)⋯*Cg*(*J*)	*X*⋯*Cg*	*Y*—*X*⋯*Cg*	*Y*⋯*Cg*
C4—O8⋯*Cg*3^i^	3.6086 (2)	68.07 (11)	3.349 (2)
C7—O11⋯*Cg*2^ii^	3.4783 (2)	68.37 (11)	3.233 (2)

Symmetry codes: (i) 1 − *x*, −*y*, −*z*; (ii) 2 − *x*, −*y*, −*z*.

**Table 4:** Experimental details

Crystal data	
Chemical formula	[CdNa_2_(C_3_H_2_N_3_O_3_)_4_(H_2_O)_8_]
*M*_r_	814.81
Crystal system, space group	Triclinic, *P*\overline{1}
Temperature (K)	293
*a*, *b*, *c* (Å)	7.0501 (3), 10.0314 (5), 19.5058 (9)
α, β, γ (°)	101.023 (1), 90.468 (1), 97.233 (1)
*V* (Å^3^)	1342.53 (11)
*Z*	2
Radiation type	Mo *K*α
μ (mm^−1^)	0.96
Crystal size (mm)	0.15 × 0.10 × 0.10

Data collection	
Diffractometer	Bruker Kappa *APEX3* CMOS
Absorption correction	Multi-scan (*SADABS*; Bruker, 2016 ▸)
*T*_min_, *T*_max_	0.870, 0.901
No. of measured, independent and observed [*I* > 2σ(*I*)] reflections	52745, 5912, 5460
*R*_int_	0.029
(sin θ/λ)_max_ (Å^−1^)	0.643

Refinement	
*R*[*F*^2^ > 2σ(*F* ^2^)], *wR*(*F* ^2^), *S*	0.024, 0.063, 1.20
No. of reflections	5912
No. of parameters	430
H-atom treatment	H-atom parameters constrained
Δρ_max_, Δρ_min_ (e Å^−3^)	0.88, −0.57

Computer programs: *APEX3*, *SAINT* and *XPREP* (Bruker, 2016 ▸), *SHELXT2014/5* (Sheldrick, 2015*a* ▸), *SHELXL2018/3* (Sheldrick, 2015*b* ▸), *ORTEP-3 for Windows* (Farrugia, 2012 ▸) and *DIAMOND* (Brandenburg, 2010 ▸).

## supplementary crystallographic information

### Crystal data

**Table:**

[CdNa_2_(C_3_H_2_N_3_O_3_)_4_(H_2_O)_8_]	*Z* = 2
*M**_r_* = 814.81	*F*(000) = 820
Triclinic, *P*1	*D*_x_ = 2.016 Mg m^−^^3^
*a* = 7.0501 (3) Å	Mo *K*α radiation, λ = 0.71073 Å
*b* = 10.0314 (5) Å	Cell parameters from 9962 reflections
*c* = 19.5058 (9) Å	θ = 3.1–29.6°
α = 101.023 (1)°	µ = 0.96 mm^−^^1^
β = 90.468 (1)°	*T* = 293 K
γ = 97.233 (1)°	Block, colourless
*V* = 1342.53 (11) Å^3^	0.15 × 0.10 × 0.10 mm

### Data collection

**Table:**

Bruker Kappa APEX3 CMOS diffractometer	5912 independent reflections
Radiation source: fine-focus sealed tube	5460 reflections with *I* > 2σ(*I*)
Graphite monochromator	*R*_int_ = 0.029
ω and φ scan	θ_max_ = 27.2°, θ_min_ = 3.2°
Absorption correction: multi-scan (SADABS; Bruker, 2016)	*h* = −9→9
*T*_min_ = 0.870, *T*_max_ = 0.901	*k* = −12→12
52745 measured reflections	*l* = −25→25

### Refinement

**Table:**

Refinement on *F*^2^	0 restraints
Least-squares matrix: full	Hydrogen site location: mixed
*R*[*F*^2^ > 2σ(*F*^2^)] = 0.024	H-atom parameters constrained
*wR*(*F*^2^) = 0.063	*w* = 1/[σ^2^(*F*_o_^2^) + (0.0277*P*)^2^ + 0.9925*P*] where *P* = (*F*_o_^2^ + 2*F*_c_^2^)/3
*S* = 1.20	(Δ/σ)_max_ = 0.001
5912 reflections	Δρ_max_ = 0.88 e Å^−^^3^
430 parameters	Δρ_min_ = −0.57 e Å^−^^3^

### Special details

**Table:**

Geometry. All esds (except the esd in the dihedral angle between two l.s. planes) are estimated using the full covariance matrix. The cell esds are taken into account individually in the estimation of esds in distances, angles and torsion angles; correlations between esds in cell parameters are only used when they are defined by crystal symmetry. An approximate (isotropic) treatment of cell esds is used for estimating esds involving l.s. planes.

### Fractional atomic coordinates and isotropic or equivalent isotropic displacement parameters (Å^2^)

**Table:**

	*x*	*y*	*z*	*U*_iso_*/*U*_eq_	
C1	0.8025 (3)	0.33005 (19)	0.28213 (10)	0.0172 (4)	
C2	0.8168 (3)	0.4798 (2)	0.39643 (11)	0.0216 (4)	
C3	0.6821 (3)	0.24087 (19)	0.37866 (10)	0.0172 (4)	
C4	0.5623 (3)	−0.32929 (19)	0.20544 (10)	0.0160 (4)	
C5	0.6255 (3)	−0.4699 (2)	0.09476 (10)	0.0194 (4)	
C6	0.6837 (3)	−0.22094 (19)	0.11527 (10)	0.0157 (4)	
C7	0.9520 (3)	0.33435 (19)	−0.20488 (10)	0.0157 (4)	
C8	0.8810 (3)	0.4855 (2)	−0.09848 (10)	0.0190 (4)	
C9	0.8168 (3)	0.23791 (19)	−0.11439 (10)	0.0158 (4)	
C10	0.1780 (3)	0.23787 (19)	0.37549 (10)	0.0166 (4)	
C11	0.3200 (3)	0.4760 (2)	0.39865 (11)	0.0213 (4)	
C12	0.3021 (3)	0.3339 (2)	0.28316 (10)	0.0170 (4)	
N1	0.7207 (2)	0.22287 (16)	0.30988 (8)	0.0169 (3)	
N2	0.7326 (3)	0.36898 (17)	0.42028 (9)	0.0226 (4)	
H2	0.709160	0.378905	0.464036	0.027*	
N3	0.8481 (3)	0.45615 (17)	0.32672 (9)	0.0215 (4)	
H3	0.899616	0.523714	0.309038	0.026*	
N4	0.6247 (2)	−0.21351 (16)	0.18213 (8)	0.0152 (3)	
N5	0.5607 (2)	−0.45516 (16)	0.16086 (8)	0.0187 (3)	
H5	0.516292	−0.527734	0.175881	0.022*	
N6	0.6887 (3)	−0.35041 (17)	0.07445 (8)	0.0195 (3)	
H6	0.734942	−0.354974	0.033533	0.023*	
N7	0.8839 (2)	0.22246 (16)	−0.17957 (8)	0.0172 (3)	
N8	0.9513 (2)	0.46392 (16)	−0.16400 (9)	0.0187 (3)	
H8	0.997548	0.533894	−0.180868	0.022*	
N9	0.8147 (3)	0.36876 (17)	−0.07555 (9)	0.0203 (4)	
H9	0.768803	0.376704	−0.034471	0.024*	
N10	0.3519 (3)	0.45707 (17)	0.32895 (9)	0.0215 (4)	
H10	0.406119	0.525452	0.312499	0.026*	
N11	0.2119 (2)	0.22607 (17)	0.30703 (9)	0.0188 (3)	
N12	0.2315 (3)	0.36306 (17)	0.41995 (9)	0.0219 (4)	
H12	0.207301	0.369553	0.463517	0.026*	
O1	0.9770 (2)	0.03762 (14)	0.20860 (7)	0.0215 (3)	
H1A	1.065349	0.092864	0.245551	0.032*	
H1B	1.036161	−0.047201	0.203744	0.032*	
O2	0.4449 (2)	0.06725 (15)	0.18140 (8)	0.0256 (3)	
H2A	0.363198	−0.014609	0.156501	0.038*	
H2B	0.391157	0.153416	0.196860	0.038*	
O3	0.8349 (2)	−0.08797 (15)	0.33509 (8)	0.0241 (3)	
H3A	0.939632	−0.020504	0.358856	0.036*	
H3B	0.901723	−0.163676	0.311768	0.036*	
O4	0.3757 (2)	−0.08155 (17)	0.30812 (8)	0.0290 (3)	
H4A	0.369042	−0.181741	0.301003	0.044*	
H4B	0.265442	−0.057541	0.284123	0.044*	
O5	0.6035 (2)	0.14863 (15)	0.40559 (8)	0.0280 (3)	
O6	0.8599 (3)	0.59241 (16)	0.43440 (8)	0.0368 (4)	
O7	0.8382 (2)	0.32046 (15)	0.21994 (8)	0.0269 (3)	
O8	0.5039 (2)	−0.32832 (15)	0.26519 (8)	0.0262 (3)	
O9	0.6280 (3)	−0.58152 (15)	0.05625 (8)	0.0312 (4)	
O10	0.7346 (2)	−0.11970 (15)	0.09073 (8)	0.0241 (3)	
O11	1.0167 (2)	0.32568 (15)	−0.26437 (8)	0.0246 (3)	
O12	0.8765 (3)	0.59950 (15)	−0.06270 (8)	0.0308 (4)	
O13	0.7540 (2)	0.13948 (15)	−0.08785 (8)	0.0236 (3)	
O14	0.1013 (2)	0.14177 (14)	0.40139 (8)	0.0227 (3)	
O15	0.3689 (3)	0.58556 (16)	0.43856 (8)	0.0353 (4)	
O16	0.3458 (2)	0.32703 (15)	0.22117 (7)	0.0248 (3)	
O17	0.5338 (2)	0.13908 (16)	0.05424 (9)	0.0313 (4)	
H17A	0.559228	0.239151	0.059155	0.047*	
H18A	1.002488	−0.103967	−0.120403	0.047*	
O18	0.9774 (2)	−0.12084 (16)	−0.07304 (8)	0.0295 (4)	
H18B	0.956220	−0.221324	−0.079922	0.044*	
H17B	0.545676	0.133976	0.104027	0.044*	
O19	0.5872 (2)	−0.13881 (16)	0.44927 (8)	0.0299 (3)	
H19A	0.537228	−0.235403	0.447693	0.045*	
H19B	0.694698	−0.133273	0.417733	0.045*	
O20	0.0908 (3)	−0.14107 (19)	0.46154 (10)	0.0411 (4)	
H20A	0.081618	−0.129379	0.512560	0.062*	
H20B	0.114905	−0.236594	0.446586	0.062*	
Na1	0.75518 (12)	0.00016 (9)	−0.00173 (4)	0.02498 (19)	
Na2	0.34777 (12)	0.00440 (9)	0.43414 (5)	0.02616 (19)	
Cd1	0.66796 (2)	0.00416 (2)	0.25149 (2)	0.01845 (5)	

### Atomic displacement parameters (Å^2^)

**Table:**

	*U*^11^	*U*^22^	*U*^33^	*U*^12^	*U*^13^	*U*^23^
C1	0.0207 (9)	0.0147 (9)	0.0163 (9)	0.0027 (7)	0.0031 (7)	0.0031 (7)
C2	0.0283 (11)	0.0171 (10)	0.0170 (10)	−0.0031 (8)	0.0054 (8)	0.0010 (8)
C3	0.0177 (9)	0.0141 (9)	0.0184 (9)	0.0006 (7)	0.0014 (7)	0.0002 (7)
C4	0.0190 (9)	0.0123 (9)	0.0156 (9)	0.0004 (7)	0.0014 (7)	0.0015 (7)
C5	0.0246 (10)	0.0157 (9)	0.0164 (9)	0.0004 (8)	0.0038 (8)	0.0011 (7)
C6	0.0175 (9)	0.0145 (9)	0.0146 (9)	0.0003 (7)	0.0000 (7)	0.0030 (7)
C7	0.0181 (9)	0.0133 (9)	0.0154 (9)	0.0020 (7)	0.0022 (7)	0.0017 (7)
C8	0.0241 (10)	0.0162 (9)	0.0158 (9)	0.0003 (8)	0.0036 (7)	0.0022 (7)
C9	0.0173 (9)	0.0137 (9)	0.0161 (9)	0.0006 (7)	−0.0005 (7)	0.0028 (7)
C10	0.0166 (9)	0.0156 (9)	0.0173 (9)	0.0020 (7)	−0.0002 (7)	0.0023 (7)
C11	0.0276 (11)	0.0175 (10)	0.0168 (9)	−0.0019 (8)	0.0051 (8)	0.0015 (8)
C12	0.0190 (9)	0.0164 (9)	0.0156 (9)	0.0033 (7)	0.0021 (7)	0.0020 (7)
N1	0.0221 (8)	0.0122 (8)	0.0150 (8)	−0.0003 (6)	0.0026 (6)	0.0011 (6)
N2	0.0357 (10)	0.0150 (8)	0.0140 (8)	−0.0052 (7)	0.0074 (7)	0.0000 (6)
N3	0.0335 (10)	0.0126 (8)	0.0170 (8)	−0.0037 (7)	0.0079 (7)	0.0032 (6)
N4	0.0220 (8)	0.0107 (7)	0.0120 (7)	−0.0003 (6)	0.0023 (6)	0.0011 (6)
N5	0.0293 (9)	0.0099 (7)	0.0156 (8)	−0.0018 (6)	0.0069 (7)	0.0021 (6)
N6	0.0301 (9)	0.0149 (8)	0.0121 (8)	−0.0006 (7)	0.0074 (7)	0.0006 (6)
N7	0.0246 (9)	0.0120 (7)	0.0151 (8)	0.0024 (6)	0.0044 (6)	0.0028 (6)
N8	0.0284 (9)	0.0109 (7)	0.0169 (8)	0.0001 (6)	0.0072 (7)	0.0040 (6)
N9	0.0307 (9)	0.0156 (8)	0.0138 (8)	0.0006 (7)	0.0088 (7)	0.0020 (6)
N10	0.0332 (10)	0.0135 (8)	0.0159 (8)	−0.0042 (7)	0.0076 (7)	0.0028 (6)
N11	0.0233 (8)	0.0152 (8)	0.0165 (8)	−0.0011 (6)	0.0017 (6)	0.0019 (6)
N12	0.0335 (10)	0.0159 (8)	0.0138 (8)	−0.0046 (7)	0.0059 (7)	0.0016 (6)
O1	0.0228 (7)	0.0180 (7)	0.0211 (7)	−0.0004 (6)	0.0020 (6)	−0.0010 (6)
O2	0.0313 (8)	0.0192 (7)	0.0241 (8)	0.0063 (6)	−0.0049 (6)	−0.0029 (6)
O3	0.0274 (8)	0.0226 (7)	0.0217 (7)	0.0048 (6)	−0.0015 (6)	0.0021 (6)
O4	0.0302 (8)	0.0309 (9)	0.0235 (8)	−0.0006 (7)	0.0018 (6)	0.0020 (7)
O5	0.0382 (9)	0.0185 (7)	0.0250 (8)	−0.0085 (6)	0.0073 (7)	0.0063 (6)
O6	0.0630 (12)	0.0175 (8)	0.0222 (8)	−0.0136 (8)	0.0136 (8)	−0.0040 (6)
O7	0.0431 (9)	0.0213 (7)	0.0149 (7)	0.0000 (7)	0.0082 (6)	0.0028 (6)
O8	0.0443 (9)	0.0171 (7)	0.0162 (7)	−0.0007 (6)	0.0125 (6)	0.0029 (6)
O9	0.0558 (11)	0.0132 (7)	0.0214 (8)	−0.0006 (7)	0.0148 (7)	−0.0019 (6)
O10	0.0374 (9)	0.0163 (7)	0.0186 (7)	−0.0023 (6)	0.0030 (6)	0.0071 (6)
O11	0.0382 (9)	0.0175 (7)	0.0180 (7)	0.0028 (6)	0.0128 (6)	0.0034 (6)
O12	0.0530 (10)	0.0135 (7)	0.0232 (8)	−0.0003 (7)	0.0145 (7)	−0.0008 (6)
O13	0.0347 (8)	0.0172 (7)	0.0190 (7)	−0.0023 (6)	0.0044 (6)	0.0071 (6)
O14	0.0279 (8)	0.0171 (7)	0.0229 (7)	−0.0046 (6)	0.0026 (6)	0.0079 (6)
O15	0.0595 (11)	0.0178 (8)	0.0212 (8)	−0.0124 (7)	0.0131 (7)	−0.0035 (6)
O16	0.0374 (9)	0.0206 (7)	0.0155 (7)	0.0013 (6)	0.0071 (6)	0.0025 (6)
O17	0.0421 (10)	0.0223 (8)	0.0290 (9)	−0.0001 (7)	0.0074 (7)	0.0066 (7)
O18	0.0414 (9)	0.0196 (8)	0.0263 (8)	−0.0002 (7)	0.0064 (7)	0.0039 (6)
O19	0.0358 (9)	0.0242 (8)	0.0292 (8)	0.0013 (7)	0.0088 (7)	0.0051 (6)
O20	0.0497 (11)	0.0333 (10)	0.0365 (10)	−0.0116 (8)	0.0067 (8)	0.0078 (8)
Na1	0.0304 (5)	0.0240 (4)	0.0226 (4)	0.0034 (4)	0.0040 (4)	0.0096 (3)
Na2	0.0257 (4)	0.0243 (4)	0.0284 (5)	−0.0008 (3)	0.0033 (3)	0.0073 (4)
Cd1	0.02259 (8)	0.01318 (8)	0.01772 (8)	0.00042 (5)	0.00074 (5)	−0.00040 (5)

### Geometric parameters (Å, º)

**Table:**

C1—O7	1.229 (2)	N6—H6	0.8600
C1—N1	1.361 (2)	N8—H8	0.8600
C1—N3	1.390 (3)	N9—H9	0.8600
C2—O6	1.227 (3)	N10—H10	0.8600
C2—N2	1.357 (3)	N12—H12	0.8600
C2—N3	1.360 (3)	O1—Cd1	2.3478 (14)
C3—O5	1.225 (2)	O1—H1A	0.9836
C3—N1	1.354 (3)	O1—H1B	0.9829
C3—N2	1.385 (2)	O2—Cd1	2.2996 (15)
C4—O8	1.238 (2)	O2—H2A	0.9828
C4—N4	1.351 (2)	O2—H2B	0.9831
C4—N5	1.388 (2)	O3—Cd1	2.3913 (15)
C5—O9	1.225 (2)	O3—H3A	0.9830
C5—N6	1.357 (3)	O3—H3B	0.9833
C5—N5	1.359 (3)	O4—Na2	2.4627 (18)
C6—O10	1.219 (2)	O4—Cd1	2.4817 (16)
C6—N4	1.363 (2)	O4—H4A	0.9830
C6—N6	1.392 (2)	O4—H4B	0.9828
C7—O11	1.242 (2)	O5—Na2	2.3067 (17)
C7—N7	1.347 (2)	O10—Na1	2.3471 (16)
C7—N8	1.389 (2)	O13—Na1	2.3824 (16)
C8—O12	1.224 (2)	O14—Na2	2.4997 (18)
C8—N9	1.363 (3)	O17—Na1	2.3584 (19)
C8—N8	1.363 (2)	O17—Na1^i^	2.4195 (19)
C9—O13	1.236 (2)	O17—H17A	0.9832
C9—N7	1.348 (2)	O17—H17B	0.9857
C9—N9	1.386 (2)	O18—Na1	2.3939 (18)
C10—O14	1.240 (2)	O18—Na1^ii^	2.4192 (19)
C10—N11	1.344 (3)	O18—H18A	0.9841
C10—N12	1.389 (3)	O18—H18B	0.9830
C11—O15	1.227 (3)	O19—Na2	2.4009 (19)
C11—N12	1.361 (3)	O19—Na2^iii^	2.4197 (18)
C11—N10	1.361 (3)	O19—H19A	0.9830
C12—O16	1.242 (2)	O19—H19B	0.9830
C12—N11	1.347 (3)	O20—Na2	2.3118 (19)
C12—N10	1.383 (3)	O20—H20A	0.9831
N1—Cd1	2.2541 (16)	O20—H20B	0.9833
N2—H2	0.8600	Na1—Na1^ii^	3.4526 (17)
N3—H3	0.8600	Na1—Na1^i^	3.5988 (17)
N4—Cd1	2.3210 (15)	Na2—Na2^iii^	3.3557 (18)
N5—H5	0.8600		
			
O7—C1—N1	123.25 (18)	C10—O14—Na2	110.29 (13)
O7—C1—N3	118.73 (18)	Na1—O17—Na1^i^	97.73 (6)
N1—C1—N3	118.01 (17)	Na1—O17—H17A	119.9
O6—C2—N2	122.93 (19)	Na1^i^—O17—H17A	124.6
O6—C2—N3	122.67 (19)	Na1—O17—H17B	104.9
N2—C2—N3	114.40 (17)	Na1^i^—O17—H17B	110.2
O5—C3—N1	122.71 (18)	H17A—O17—H17B	98.3
O5—C3—N2	118.59 (18)	Na1—O18—Na1^ii^	91.67 (6)
N1—C3—N2	118.70 (17)	Na1—O18—H18A	120.5
O8—C4—N4	122.70 (17)	Na1^ii^—O18—H18A	107.0
O8—C4—N5	117.98 (17)	Na1—O18—H18B	116.6
N4—C4—N5	119.31 (17)	Na1^ii^—O18—H18B	118.1
O9—C5—N6	122.21 (18)	H18A—O18—H18B	103.3
O9—C5—N5	123.24 (18)	Na2—O19—Na2^iii^	88.23 (6)
N6—C5—N5	114.55 (17)	Na2—O19—H19A	114.5
O10—C6—N4	122.79 (17)	Na2^iii^—O19—H19A	114.5
O10—C6—N6	119.38 (17)	Na2—O19—H19B	114.5
N4—C6—N6	117.82 (16)	Na2^iii^—O19—H19B	114.5
O11—C7—N7	121.84 (17)	H19A—O19—H19B	109.5
O11—C7—N8	118.25 (17)	Na2—O20—H20A	109.9
N7—C7—N8	119.91 (17)	Na2—O20—H20B	109.5
O12—C8—N9	122.17 (18)	H20A—O20—H20B	104.2
O12—C8—N8	123.57 (18)	O10—Na1—O17	88.93 (6)
N9—C8—N8	114.26 (17)	O10—Na1—O13	173.39 (7)
O13—C9—N7	122.52 (18)	O17—Na1—O13	84.51 (6)
O13—C9—N9	118.21 (17)	O10—Na1—O18	100.11 (6)
N7—C9—N9	119.27 (17)	O17—Na1—O18	170.94 (7)
O14—C10—N11	123.13 (18)	O13—Na1—O18	86.45 (6)
O14—C10—N12	117.85 (17)	O10—Na1—O17^i^	89.31 (6)
N11—C10—N12	119.02 (17)	O17—Na1—O17^i^	82.27 (6)
O15—C11—N12	123.32 (19)	O13—Na1—O17^i^	90.60 (6)
O15—C11—N10	122.43 (19)	O18—Na1—O17^i^	97.21 (6)
N12—C11—N10	114.24 (17)	O10—Na1—O18^ii^	78.82 (6)
O16—C12—N11	122.52 (18)	O17—Na1—O18^ii^	93.97 (6)
O16—C12—N10	117.89 (18)	O13—Na1—O18^ii^	100.80 (6)
N11—C12—N10	119.58 (17)	O18—Na1—O18^ii^	88.33 (6)
C3—N1—C1	120.31 (16)	O17^i^—Na1—O18^ii^	167.64 (7)
C3—N1—Cd1	114.93 (12)	O10—Na1—Na1^ii^	89.19 (5)
C1—N1—Cd1	124.46 (13)	O17—Na1—Na1^ii^	137.16 (6)
C2—N2—C3	124.18 (17)	O13—Na1—Na1^ii^	95.07 (5)
C2—N2—H2	117.9	O18—Na1—Na1^ii^	44.46 (4)
C3—N2—H2	117.9	O17^i^—Na1—Na1^ii^	140.49 (6)
C2—N3—C1	124.38 (17)	O18^ii^—Na1—Na1^ii^	43.88 (4)
C2—N3—H3	117.8	O10—Na1—Na1^i^	88.84 (5)
C1—N3—H3	117.8	O17—Na1—Na1^i^	41.77 (4)
C4—N4—C6	120.03 (16)	O13—Na1—Na1^i^	86.81 (5)
C4—N4—Cd1	124.47 (12)	O18—Na1—Na1^i^	137.02 (6)
C6—N4—Cd1	115.24 (12)	O17^i^—Na1—Na1^i^	40.49 (4)
C5—N5—C4	123.55 (16)	O18^ii^—Na1—Na1^i^	134.60 (6)
C5—N5—H5	118.2	Na1^ii^—Na1—Na1^i^	177.78 (5)
C4—N5—H5	118.2	O5—Na2—O20	179.41 (8)
C5—N6—C6	124.54 (16)	O5—Na2—O19	83.92 (6)
C5—N6—H6	117.7	O20—Na2—O19	96.15 (7)
C6—N6—H6	117.7	O5—Na2—O19^iii^	83.78 (6)
C7—N7—C9	119.30 (16)	O20—Na2—O19^iii^	96.81 (7)
C8—N8—C7	123.21 (16)	O19—Na2—O19^iii^	91.77 (6)
C8—N8—H8	118.4	O5—Na2—O4	77.17 (6)
C7—N8—H8	118.4	O20—Na2—O4	102.25 (7)
C8—N9—C9	124.01 (17)	O19—Na2—O4	85.43 (6)
C8—N9—H9	118.0	O19^iii^—Na2—O4	160.92 (7)
C9—N9—H9	118.0	O5—Na2—O14	94.52 (6)
C11—N10—C12	123.56 (17)	O20—Na2—O14	85.34 (7)
C11—N10—H10	118.2	O19—Na2—O14	172.40 (7)
C12—N10—H10	118.2	O19^iii^—Na2—O14	95.47 (6)
C10—N11—C12	119.66 (17)	O4—Na2—O14	86.97 (6)
C11—N12—C10	123.89 (17)	O5—Na2—Na2^iii^	81.14 (5)
C11—N12—H12	118.1	O20—Na2—Na2^iii^	99.33 (6)
C10—N12—H12	118.1	O19—Na2—Na2^iii^	46.11 (4)
Cd1—O1—H1A	110.2	O19^iii^—Na2—Na2^iii^	45.65 (5)
Cd1—O1—H1B	110.1	O4—Na2—Na2^iii^	128.64 (6)
H1A—O1—H1B	97.0	O14—Na2—Na2^iii^	141.06 (6)
Cd1—O2—H2A	109.8	N1—Cd1—O2	88.83 (6)
Cd1—O2—H2B	119.7	N1—Cd1—N4	174.58 (6)
H2A—O2—H2B	120.2	O2—Cd1—N4	88.80 (5)
Cd1—O3—H3A	111.0	N1—Cd1—O1	87.44 (5)
Cd1—O3—H3B	110.8	O2—Cd1—O1	111.45 (5)
H3A—O3—H3B	103.1	N4—Cd1—O1	88.89 (5)
Na2—O4—Cd1	117.77 (6)	N1—Cd1—O3	95.46 (6)
Na2—O4—H4A	107.4	O2—Cd1—O3	166.49 (5)
Cd1—O4—H4A	107.4	N4—Cd1—O3	87.94 (5)
Na2—O4—H4B	107.3	O1—Cd1—O3	81.59 (5)
Cd1—O4—H4B	107.3	N1—Cd1—O4	100.77 (6)
H4A—O4—H4B	109.5	O2—Cd1—O4	81.82 (6)
C3—O5—Na2	155.60 (16)	N4—Cd1—O4	83.72 (5)
C6—O10—Na1	152.13 (14)	O1—Cd1—O4	164.73 (5)
C9—O13—Na1	153.27 (14)	O3—Cd1—O4	84.79 (5)
			
O5—C3—N1—C1	177.79 (19)	O11—C7—N7—C9	179.44 (18)
N2—C3—N1—C1	−1.9 (3)	N8—C7—N7—C9	−0.5 (3)
O5—C3—N1—Cd1	−8.2 (3)	O13—C9—N7—C7	−179.33 (19)
N2—C3—N1—Cd1	172.08 (14)	N9—C9—N7—C7	1.7 (3)
O7—C1—N1—C3	−178.84 (19)	O12—C8—N8—C7	−178.5 (2)
N3—C1—N1—C3	1.4 (3)	N9—C8—N8—C7	1.4 (3)
O7—C1—N1—Cd1	7.8 (3)	O11—C7—N8—C8	178.91 (19)
N3—C1—N1—Cd1	−172.02 (13)	N7—C7—N8—C8	−1.2 (3)
O6—C2—N2—C3	179.9 (2)	O12—C8—N9—C9	179.9 (2)
N3—C2—N2—C3	0.4 (3)	N8—C8—N9—C9	0.0 (3)
O5—C3—N2—C2	−178.7 (2)	O13—C9—N9—C8	179.48 (19)
N1—C3—N2—C2	1.0 (3)	N7—C9—N9—C8	−1.5 (3)
O6—C2—N3—C1	179.5 (2)	O15—C11—N10—C12	178.5 (2)
N2—C2—N3—C1	−0.9 (3)	N12—C11—N10—C12	−0.8 (3)
O7—C1—N3—C2	−179.7 (2)	O16—C12—N10—C11	−176.9 (2)
N1—C1—N3—C2	0.1 (3)	N11—C12—N10—C11	1.9 (3)
O8—C4—N4—C6	−178.62 (19)	O14—C10—N11—C12	−177.67 (19)
N5—C4—N4—C6	0.6 (3)	N12—C10—N11—C12	2.1 (3)
O8—C4—N4—Cd1	7.4 (3)	O16—C12—N11—C10	176.32 (19)
N5—C4—N4—Cd1	−173.39 (13)	N10—C12—N11—C10	−2.5 (3)
O10—C6—N4—C4	177.24 (19)	O15—C11—N12—C10	−179.0 (2)
N6—C6—N4—C4	−4.1 (3)	N10—C11—N12—C10	0.3 (3)
O10—C6—N4—Cd1	−8.3 (2)	O14—C10—N12—C11	178.8 (2)
N6—C6—N4—Cd1	170.42 (13)	N11—C10—N12—C11	−1.0 (3)
O9—C5—N5—C4	178.2 (2)	N1—C3—O5—Na2	−79.6 (4)
N6—C5—N5—C4	−1.6 (3)	N2—C3—O5—Na2	100.1 (4)
O8—C4—N5—C5	−178.30 (19)	N4—C6—O10—Na1	−143.5 (2)
N4—C4—N5—C5	2.5 (3)	N6—C6—O10—Na1	37.8 (4)
O9—C5—N6—C6	178.0 (2)	N7—C9—O13—Na1	118.8 (3)
N5—C5—N6—C6	−2.3 (3)	N9—C9—O13—Na1	−62.3 (4)
O10—C6—N6—C5	−176.13 (19)	N11—C10—O14—Na2	87.6 (2)
N4—C6—N6—C5	5.1 (3)	N12—C10—O14—Na2	−92.13 (18)

Symmetry codes: (i) −*x*+1, −*y*, −*z*; (ii) −*x*+2, −*y*, −*z*; (iii) −*x*+1, −*y*, −*z*+1.

### Hydrogen-bond geometry (Å, º)

**Table:**

*D*—H···*A*	*D*—H	H···*A*	*D*···*A*	*D*—H···*A*
O1—H1*A*···N11^iv^	0.98	1.82	2.788 (2)	168
O1—H1*B*···N7^ii^	0.98	1.89	2.860 (2)	169
N2—H2···O15^v^	0.86	1.96	2.817 (2)	173
O2—H2*A*···O13^i^	0.98	1.77	2.716 (2)	161
O2—H2*B*···N11	0.98	2.53	3.236 (2)	128
O2—H2*B*···O16	0.98	1.79	2.753 (2)	167
N3—H3···O11^vi^	0.86	1.92	2.773 (2)	173
O3—H3*A*···O14^iv^	0.98	1.91	2.871 (2)	166
O3—H3*B*···O11^ii^	0.98	1.87	2.842 (2)	169
N5—H5···O16^vii^	0.86	2.10	2.938 (2)	164
N6—H6···O12^vii^	0.86	2.13	2.979 (2)	168
N10—H10···O8^viii^	0.86	1.94	2.792 (2)	172
N8—H8···O7^vi^	0.86	2.02	2.862 (2)	167
N12—H12···O6^v^	0.86	2.03	2.882 (2)	173
N9—H9···O9^viii^	0.86	2.04	2.885 (2)	169
O20—H20*B*···O6^ix^	0.98	2.30	2.903 (2)	119
O20—H20*A*···O14^x^	0.98	2.14	3.008 (2)	147
O4—H4*A*···O8	0.98	1.88	2.725 (2)	143
O17—H17*B*···O2	0.99	1.87	2.768 (2)	149
O17—H17*A*···O9^viii^	0.98	1.81	2.790 (2)	171
O18—H18*B*···O12^vii^	0.98	1.91	2.852 (2)	160

Symmetry codes: (i) −*x*+1, −*y*, −*z*; (ii) −*x*+2, −*y*, −*z*; (iv) *x*+1, *y*, *z*; (v) −*x*+1, −*y*+1, −*z*+1; (vi) −*x*+2, −*y*+1, −*z*; (vii) *x*, *y*−1, *z*; (viii) *x*, *y*+1, *z*; (ix) *x*−1, *y*−1, *z*; (x) −*x*, −*y*, −*z*+1.
